# Role and Merits of Green Based Nanocarriers in Cancer Treatment

**DOI:** 10.3390/cancers13225686

**Published:** 2021-11-13

**Authors:** Abdulrahman M. Elbagory, Rahaba Makgotso Marima, Zodwa Dlamini

**Affiliations:** SAMRC Precision Oncology Research Unit (PORU), Pan African Cancer Research Institute (PACRI), University of Pretoria, Pretoria 0028, South Africa; rahaba.marima@up.ac.za

**Keywords:** green nanotechnology, nanocarriers, drug delivery system, plant extracts, polysaccharides nanoparticles, microorganism-based nanoparticles, anticancer activity

## Abstract

**Simple Summary:**

The use of chemotherapy drugs against tumours is associated with various drawbacks such as poor solubility, low stability, high toxicity, lack of selectivity and rapid clearance. Nanocarriers can improve the safety and efficiency of drugs by increasing their solubility, enhance their circulation time and improve their uptake into cancer cells. Natural materials can be incorporated in the fabrication of nanocarriers as a substitute to synthetic ingredients. Several studies developed different types of green based nanocarriers using materials obtained from plant or microbial sources such as polysaccharides and polyphenols without the need of toxic chemicals in the synthesis. The green components can have many roles for example as mechanical support, trigger pH response for drug release, or act as a targeting ligand. The inclusion of these green components will support the cost effective and feasible large-scale production of nanocarriers with minimum negative impact on the environment.

**Abstract:**

The use of nanocarriers for biomedical applications has been gaining interests from researchers worldwide for the delivery of therapeutics in a controlled manner. These “smart” vehicles enhance the dissolution and the bioavailability of drugs and enable their delivery to the target site. Taking the potential toxicity into consideration, the incorporation of natural “green” materials, derived from plants or microbial sources, in the nanocarriers fabrication, improve their safety and biocompatibility. These green components can be used as a mechanical platform or as targeting ligand for the payload or can play a role in the synthesis of nanoparticles. Several studies reported the use of green based nanocarriers for the treatment of diseases such as cancer. This review article provides a critical analysis of the different types of green nanocarriers and their synthesis mechanisms, characterization, and their role in improving drug delivery of anticancer drugs to achieve precision cancer treatment. Current evidence suggests that green-based nanocarriers can constitute an effective treatment against cancer.

## 1. Introduction

Nanotechnology is an interdisciplinary field of science concerned with the processing of matter at atomic/molecular level [1]. Nanotechnology is applicable in many areas such as the chemical industry, pharmaceutical industry, optics, electronics, energy science and biomedical sciences [2]. The size of the nanoparticles (NPs) is comparable to the size of proteins and various intracellular macromolecules, which allows them to take advantage of cellular machinery to assist in the drug delivery [3]. Additionally, in comparison to bulky materials, NPs have large surface to volume ratio allowing for chemical modifications to tune their properties [4]. NPs can be used as nanocarriers to encapsulate drugs or biomolecules inside their structures and/or absorb them on their surfaces. There are different types of nanocarriers including polymeric NPs, liposomes, micelles, dendrimer, hydrogel, mesoporous, 0, 1 and 2-D materials [5]. These types are broadly divided into organic, inorganic and hybrid nanocarriers [6]. Several types of payloads can be delivered using NPs such as conventional drugs, polypeptides, proteins, vaccines, nucleic acids, genes, etc. [7].

The commonly used conventional chemotherapies include alkylating agents, antitumour antibiotics (e.g., epirubicin, doxorubicin (DOX)), antimetabolites (e.g., 5-fluorouracil (5-FU), methotrexate, gemcitabine), topoisomerase inhibitors, and mitotic inhibitors (e.g., paclitaxel and docetaxel) [8]. Chemotherapy drug delivery for local and metastatic tumours is associated with various drawbacks. Such problems may include high toxicity in normal cells, lack of tumour target selectivity, high volume drug distribution and rapid drug clearance [9]. Nanocarriers can improve the safety and efficiency of drugs by increasing their water solubility and stability, enhance their circulation time, improve their uptake by targeted cancer cells or prevent their enzyme degradation [10]. The current reports on the use of nanocarriers for drug delivery focus on: (1) the choice of suitable carrier materials to achieve high drug encapsulation rate and controlled and targeted release speed; (2) improvement of targeting ability via surface functionalization; (3) augmentation of drug biological activity with using carrier materials of similar activity; (4) formulating responsive nanocarriers that are able to release the loaded drugs at designated sites in a response to the local environment (e.g., pH-response release and response to enzymatic degradation of nanocarriers, etc.); (5) performing in vitro and in vivo assays to compare the biological activity between the loaded drugs and their free forms and to assess the safety of the nanocarriers and their stability. The use of nanocarriers inside the body will allow them to interact with blood components and vessels, normal tissues, etc., meaning that they can influence human health and therefore it is important to consider the safety of the components included in the synthesis of these nanomaterials. Despite advancements in nanocarriers, their transformation in medical applications remains insufficient. This mainly is due to their lack of biodegradation, instability in circulation, poor bioavailability, long-term potential toxicity, and inadequate tissue distribution.

Therefore, the overall aim of including green components in the fabrication of NPs as nanocarriers is to decrease toxicity to the body, to have an environmentally benign industry process and to increase affordability. The green synthesis may be facilitated by plants extracts and microbes or their isolated biomolecules [11]. The green based nanocarriers are mainly synthesized by the bottom-up approach within three fundamental conditions of synthesis. These conditions are based on the selection of a green non-toxic solvent, coupled by a good reducing agent, and thirdly incorporating an efficient stabilization material.

In this review, we will highlight different examples of green based nanocarriers used in the drug delivery of conventional anticancer drugs that contain a plant or microbial-derived components in their formulation. The review will highlight the role of the different green component used in the synthesis or in the drug delivery mechanism. In addition to the safety of using these green components, the review will also include the various benefits and the advantages of using each type of the green components.

## 2. Green Components in Nanocarriers for Anticancer Therapy

### 2.1. Polysaccharides-Based Nano Delivery Systems

Unlike petroleum-based polymers, the natural biopolymers are extensively used in agricultural, environmental and medical industries due to their nontoxic and sustainable properties [12]. An example of biopolymeric compounds are polysaccharides which are polymers of monosaccharides connected via glycosidic bonds and can be acquired from animals (e.g., chitosan, chondroitin), plants (e.g., cellulose), seaweed (e.g., carrageenan (CR)) and microorganisms (e.g., alginate, pullulan) [13,14]. Chemically, polysaccharides differ in charge, chain length, the sequence of the monosaccharides and stereochemistry, which render them useful in the preparation of functionalized materials for biomedical applications [15]. Polysaccharides can reduce and stabilize metal salts for the synthesis of metallic nanoparticles (MNPs). In addition, the presence of hydrophilic functional groups (e.g., -OH, -COOH, -NH_2_) in their structures enables them to establish non-covalent bonds with biological cells and to form bioadhesive polymers that can improve the efficacy of different drugs [15]. Polysaccharides also play an important role in cell-to-cell recognition and have selectivity to more than one site, which makes them useful in formulating drug delivery systems against multi-gene and multi-step diseases such as cancer.

Chen et al. (2019) used CR oligosaccharides (obtained from the hydrolysis of CR) as bio reducing and stabilizing material for the synthesis of spherical gold nanoparticles (GNPs). The negatively charged NPs were used as carriers for the positively charged anticancer drug, epirubicin. The (CR oligosaccharides/GNPs/epirubicin) nanosystem showed significantly lower IC_50_ value (0.087 ± 0.036 μmol/L) than the free epirubicin (0.173 ± 0.043 μmol/L) against human liver cancer cells (HepG2) as determined by the sulforhodamine B colorimetric assay. The average size of this nanocarrier was found to be 141 ± 6 nm, which could facilitate the uptake of the nanocarrier in cancer cells via endocytosis. The confocal imaging technique confirmed the uptake of the loaded epirubicin in the DAPI stained human colon cancer cells (HCT-116) as shown by the presence of red fluorescence in the cytoplasm and the nucleus. Interestingly, the red fluorescence produced after incubating the cells with the nanocarrier was more intense than the red fluorescence formed from the free epirubicin, indicating higher uptake of the loaded epirubicin [16]. The work done by Pavli et al. (2011) also exploited the electrostatic interaction between the anionic CR and the cationic properties of another anticancer drug, doxazosin [17].

One study explored the use of magnetic CR and chitosan as a hydrogel nanocomplex for the drug delivery for methotrexate [18]. The authors first synthesized iron oxide nanoparticles (Fe_3_O_4_ NPs) in the presence of CR. The produced magnetic CR was then allowed to perform electrostatic bond with protonated chitosan to give a hydrogel. The swelling studies showed that water absorbency of the hydrogel was decreased by the presence of magnetic NPs and by increasing the amount of chitosan. This was attributed to the hydrogen bonding between the Fe_3_O_4_ NPs and the CR/chitosan complex and to the increase in the electrostatic interactions between the complex components. The magnetic hydrogel was then utilized as a carrier for methotrexate. The encapsulation of methotrexate was higher by over 60% when increasing the magnetic NPs and chitosan contents. The drug release study was done in different pH buffers that mimic the blood (pH 7.4) and intracellular conditions of cancerous cells (pH 5.3). Methotrexate showed higher release at pH 7.4 (almost 70%) after 4 hr, and slower release (around 50%) at pH 5.3 at the same time.

**Figure 1:**
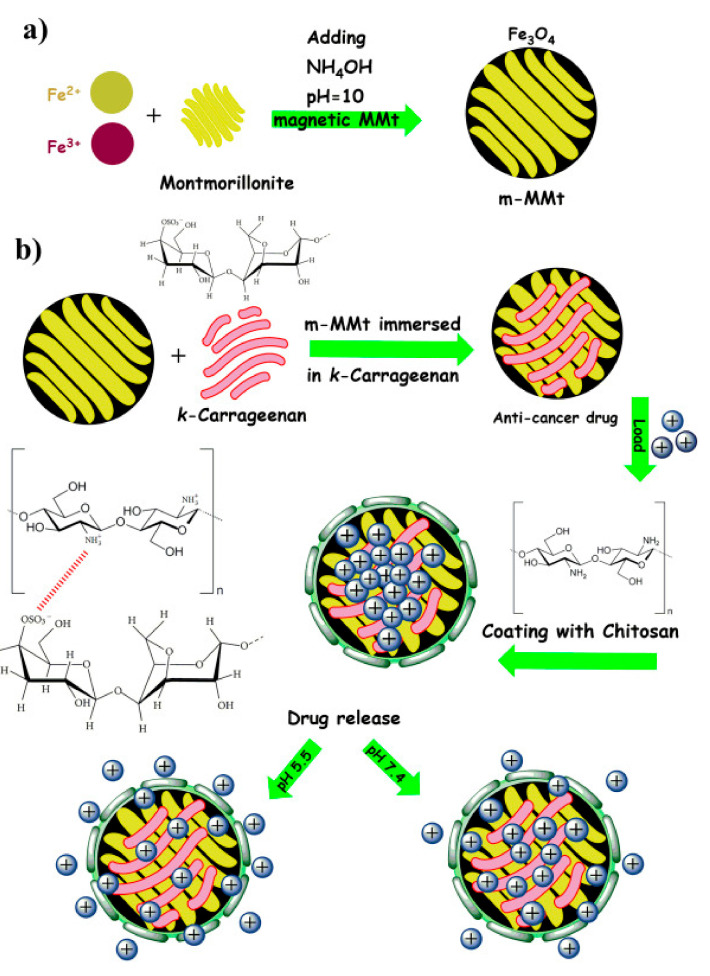
A diagram showing the synthesis of m-MMt (**a**), and the m-MMt/CR/chitosan hydrogel (**b**). Reproduced from [19].

Jafari et al. (2021) performed a similar work of crosslinking the chitosan and CR but with incorporating nanoplates of magnetic montmorillonite (m-MMt) to acquire more mechanically stable hydrogel with enhanced drug release control in order to minimize the adverse effects of anticancer drugs (Figure 1). CR was allowed to form electrostatic bond with the m-MMt, the mixture was loaded with sunitinib as an anticancer drug model. The resulted composite was later coated with chitosan facilitated by the interaction between the protonated amine group of chitosan with the anionic sulphate group of CR. The loading of sunitinib and its controlled released was found to be improved by increasing the amount of m-MMt used. The reason for this is the strong interaction between the cationic nature of sunitinib and the anionic core of the m-MMt as well as the high surface area of the m-MMt [19].

Yew et al. (2020) combined the solutions of CR (as a stabilizer), montmorillonite (MMt) (as a mechanical support) and Fe^3+^/Fe^2+^ chloride salts in an alkaline environment to formulate CR/MMt/ F_3_O_4_ NPs nanocomposite. The anticancer drug, protocatechuic acid, was loaded into the nanocomposite and exhibited release rate of 92% at pH 4.8 and 15% at pH 7.4. The free drug had a release rate higher than 85% at both pH levels, which shows the potential targeting effect of this nanocomposite [20].

The use of plant derived gum in the synthesis of NPs was also studied for their biocompatibility and non-toxic nature as opposed to the organic solvents. Gum karaya, obtained from the *Sterculia* plant species, was reported in the reduction and stabilization of GNPs in which several reaction parameters such as time, temperature, gum and gold salt concentrations and ratios were selected to obtain NPs with desired physicochemical properties. The results from the MTT assay on human lung cancer cells (A549) showed that the gum karaya/GNPs loaded with gemcitabine hydrochloride induced lower cell viability (35.1% at 1.0 µg/mL) than the free drug at the same concentration (46.8%). It has been shown that the anticancer mode of action of gemcitabine hydrochloride includes the ROS generation in the cancer cells [21]. The authors found that the ROS generation levels were significantly higher after treating the cells with the loaded gemcitabine hydrochloride in comparison to its free form [22].

Godugu and beero (2018) loaded 5-FU into platinum nanoparticles (PtNPs) synthesized and stabilized using gum kondagogu, a natural anionic gum from the Indian *Cochlospermum Gossypium* (Family: Bixaceae). The authors observed the UV-Vis of the gum kondagogu and platinum salt solution mixtures at different conditions and selected pH 8.0 and heating at 100 °C as the best reaction parameters. Further, the NPs exhibited sustained release of the 5-FU. The authors used dialysis bag to measure the drug release % and the HPLC profiling indicated the sustained release of the loaded 5-FU (67.3 ± 0.2%) compared to 92.9 ± 0.5% of the free 5-FU in 120 min. The authors also utilized the *Agrobacterium tumefaciens*-induced potato disc tumour assay to evaluate the cytotoxicity of the loaded and free 5-FU. The 5-FU loaded in the nanocarrier showed complete tumour inhibition at concentration of 30 µg/mL, whereas 60 µg/mL of the free 5-FU was needed to completely inhibit the tumour [23].

**Figure 2:**
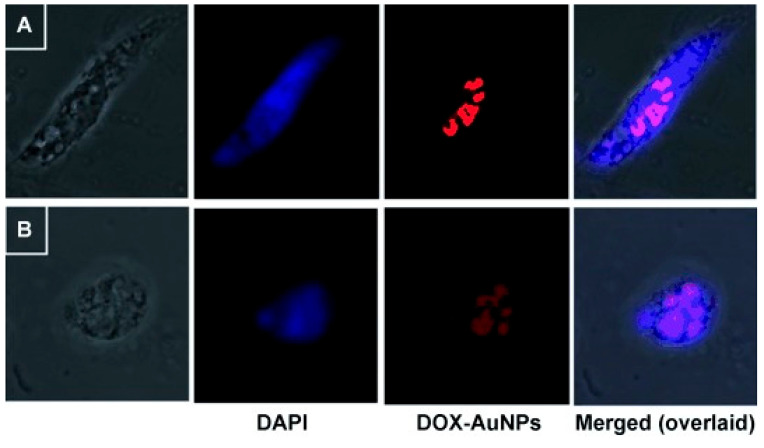
Confocal microscopy images showing DAPI stained cells ((**A**) LN-18 and (**B**) LN-229) demonstrating the apoptotic effects of DOX loaded on the gum-based GNPs (denoted DOX-AuNPs). Reproduced from [25].

The bacterial derived gellan gum is another anionic polysaccharide that was embedded with a silkworm protein, sericin, and the rice bran albumin to form a green protein-polysaccharide nanocomposite which was loaded with DOX [24]. This nanocomposite was pH responsive due to the presence of sericin while the rice bran albumin was used to release the loaded DOX in a controlled matter. The nanocomposite showed a swilling rate of 72% and 32% after 40 h at acidic and basic pH, respectively. This shows the ability of the nanocomposite to induce sustained release in the tumour environment [24]. A previous work utilized the reducing properties gellan gum to synthesize GNPs which did not show any significant UV-Vis alterations under wide range of pH (3.0–10) and electrolyte conditions (NaCl 0.1 M). The gum-based GNPs were loaded with DOX, which showed significant cytotoxicity against glioma cell lines (LN-18 and LN-229) more than the free DOX. The confocal microscopy images showed that the DOX loaded on the gum-based GNPs induced cell death via apoptosis (Figure 2) [25].

Maltodextrin, an (1,4)-glucose polymer, is water soluble and can be used as hydrophilic carrier for pharmaceutical active ingredients, and can be obtained by the partial hydrolysis of starch [26]. A study reported the preparation of polysaccharide NPs composed of gum Arabic and maltodextrin functionalized with epigallocatechin-3-gallate (EGCG). It was demonstrated by the clonogenic assay that encapsulated EGCG had enhanced inhibitory effect on human androgen independent prostate cancer cells (DU145) at lower concentrations compared to the pristine EGCG [27].

Porphyran is a linear sulphated polysaccharide obtained from the red algae *Porphyra* sp. with reported anticancer properties [28,29]. The synthesis of GNPs using porphyran as a reducing and capping agent was done at pH 11 and with heating at 70 °C. DOX was loaded on the produced GNPs leading to the increase of the zeta potential due to the positive charge of DOX. The cytotoxicity evaluation against LN-229 showed that the loaded DOX was four times toxic than its free form, which was attributed to the increased uptake of the loaded DOX via endocytosis. Further, the porphyran GNPs enabled controlled of the payload with the amount of DOX released was six times at acidic pH compared to the basic pH condition [30].

Several research articles reported the use of cyclodextrin (CD) in the formation of polysaccharides-based NPs. CD (also known as macrocyclic receptors) are key players in drug delivery because they can bind non-covalently to drugs, are biocompatible and have high resistance to non-enzymatic degradation with high thermal and chemical stability, making them particularly useful for drug delivery through oral routes [31].

Zhang et al. (2014) used CD and graphene oxide nanosheets as a scaffold to load hyaluronated adamantane (as a hyaluronic acid (HA) receptor targeting ligand) and the poorly soluble anticancer, camptothecin. The authors factionalized the graphene oxide sheets with CD using amine-epoxy reaction before loading the targeting ligand and camptothecin non-covalently. The in vitro cytotoxicity assay revealed that the tertiary assembly induced higher cytotoxicity against MDA-MB-231 (human breast cancer cells with high expression of HA receptors) with around 51.5% viability compared to 64.5% viability recorded after incubation the cells with the free camptothecin. The cytotoxicity of the nanocomposite was lower (82.5%) after the incubation with normal fibroblast cells compared to the free drug (63.0%) indicating the selectivity of nanocomposite towards the cancer cells [32].

The size and crystallinity of the CD metal-framework were optimized for the first-time using seed-mediated synthesis with starch NPs and also by controlling incubation time. This framework showed relatively small in vitro cytotoxicity and were also used to increase the stability and induced controlled release of resveratrol [33].

The natural and non-immunogenic, alginate, is readily available and biodegradable anionic polymer [34]. The backbone of this marine algae isolate has hydroxyl groups that can reduce gold salts into GNPs. Additionally, the carboxylic groups found in its structure can play a role in the stabilization of the GNPs [35].

**Figure 3:**
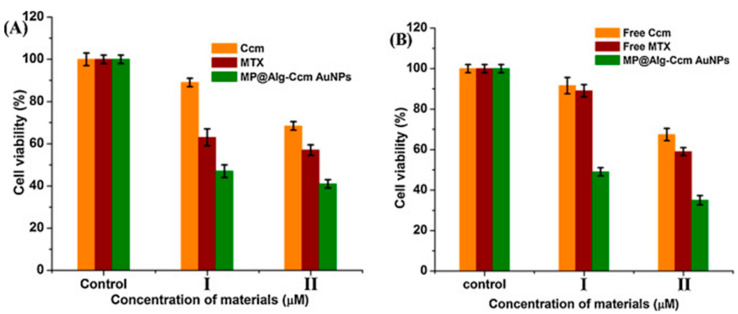
The effect of free curcumin (denoted Ccm) and the loaded curcumin on the nanocarrier (denoted MP@Alg-Ccm AuNPs) of the cell viability of (**A**) C6 and (**B**) MCF-7 cells as established by the MTT assay. Reproduced from [36].

Dey et al. (2016) used alginate and curcumin conjugate to synthesize and stabilize GNPs and was subsequently loaded with methotrexate covalently after the conjugation with bis(aminopropyl) terminated polyethylene glycol (PEG). This dual drug (curcumin and methotrexate) biopolymer showed enhanced cytotoxicity effect against rat glioma (C6) and human breast cancer (MCF-7) cell lines in comparison to the free drugs. The authors related this enhanced activity of the biopolymer to its suitable hydrodynamic size (187 nm), the negatively charged GNPs and the hydrophilic corona protection (alginate and PEG) that enabled the NPs to avoid reticuloendothelial system clearance and acquire enhanced permeation and retention effect (Figure 3) [36].

Pullulan is another polysaccharide that is produced by the *Aureobasidium pullulans* fungus [37]. The low viscosity and high-water solubility of this polymer give it an advantage over other polysaccharides (e.g., CR, chitosan and cellulose) that would require further derivatization making them unsuitable for large-scale synthesis [38].

A study reported an easy methodology of the biosynthesis of GNPs from alkali treated pullulan with the aid of its hydroxyl groups. The authors optimized different reaction parameters to control the morphology of the biocompatible GNPs. The study also reported the loading two cassiarin A chloride derivatives as anticancer drug models which showed higher toxicity against human gastric carcinoma cells (KATO-III) compared to their free forms and the positive control cisplatin [39]. The same authors also used a derivatised pullulan (with *para*-aminobenzoic acid-quat188-pullulan) for the synthesis of GNPs. This functionalization allowed a better intramolecular interaction and stronger interaction with negative charged cancer cell facilitated by the positively charged quat188 and hydrophobic attraction of *para*-aminobenzoic acid with the hydrophobic sections of the cells. This was demonstrated by the two times higher cytotoxicity of the DOX loaded into these GNPs compared to the free DOX against Chago cells [40]. In a continuation of the same work, the authors decorated the same DOX loaded Pullulan derivatised GNPs with folate (FA) to enhance its targeting effect, which yielded around five-fold higher cytotoxicity compared to the pristine drug against Chago cells. This increase in the cytotoxicity was mainly attributed to the targeting effect of FA to the overexpressed folate receptors on the Chago cells [41].

Carboxymethylcellulose (CMC) is a water-soluble anionic biopolymer cellulose derivative that is non-hazardous, biodegradable, and renewable with pH-sensitivity making it excellent for drug delivery systems [42].

A nanocomposite composed of CMC/Zinc/graphene oxide was synthesized with a facile one-pot method using solvothermal technique. The nanocomposite was loaded with DOX and showed minimal release of the drug under normal physiological conditions. Interestingly, the drug release of DOX from graphene oxide nanosheets without the CMC/Zinc framework was higher under the same conditions. which shows the potential of the nanocomposite to minimize the side effects of DOX on normal tissues. Conversely, the release of DOX at pH 5 from the nanocomposite was higher than its release from the graphene oxide alone, citing the role of nanocomposite in minimizing the side effects of DOX against normal tissue and in increasing its targeting effect against cancer tissue. Similarly, the cytotoxicity of CMC/Zinc/graphene oxide nanocomposite loaded with DOX was higher than graphene oxide loaded with DOX against human blood cancer cell line (K562). This increased cytotoxicity was explained by the positive charge of the nanocomposite, which can be attracted to the cancer cells. However, the free DOX induced higher cytotoxicity than the loaded DOX which the authors contributed to the sustained release from the nanocomposite [43].

Sun et al. (2019) reported the formation of CMC and chitosan pH responsive nanocomposite in order to eliminate their individual limitations in delivering drugs in gastrointestinal environment. The nanocomposite was also coupled with zinc oxide nanoparticles (ZnONPs) to assist in burst release prevention. The 5-FU was used as a drug model, which exhibited greater adsorption into the nanocomposite by increasing the CMC quantity because of the attraction between the carboxyl groups of CMC and the hydroxyl and amine groups of 5-FU. The swelling rate was found to be minimum at pH 1.2 making the nanocomposite suitable for oral drug delivery in the treatment of colon cancer [44]. Pooresmaeil et al. (2019) also used CMC to prevent the gastric burst release of 5-FU entrapped in layered double hydroxide of zinc and aluminium [45].

CMC was also used for the first time to cap fluorescent quantum dots composed of Cu-In-S/ZnS for theranostic purposes. In the following, the CMC stabilized quantum dots were functionalized with FA and DOX. The cytotoxicity assays recorded lower antiproliferative properties against the FRα—HEK 293T and MCF-7 cells compared to the unbound DOX. This was attributed to the sustained release by the polymeric quantum dots that can be taken up by the cells via endocytosis, whereas the free drug can also infiltrate the cells by diffusion. However, the loaded DOX showed 30% higher cytotoxicity than the free DOX towards the FRα + TNBC cells, facilitated by the presence of FA in the quantum dots. The confocal laser microscopy images revealed the DOX was mainly internalized in the nucleus causing cell death by intercalating with the DNA and affecting the cell metabolism [46]. Similar work also used CMC as a stabilizer for Bi_2_O_2_CO_3_ quantum dots that were used to load sorafenib for liver cancer drug delivery. The dialysis analysis showed over 77% drug release in case of loaded sorafenib and only 2.47% drug was released from the sorafenib control solution [47].

### 2.2. Plant Extracts-Based Nanocarriers

The use of plant extracts in the biosynthesis of different types of NPs has been widely explored due to their availability, renewability, easy and safe handling compared to other green routes such as bacteria or fungi. The presence of effective phytochemicals with different functional groups such as ketones, aldehydes, flavones, amides, terpenoids, carboxylic acids, phenols, and saponins can reduce metal salts into MNPs without the need for chemical stabilizers/capping agents.

Biodegradable poly(D,L-lactide) NPs were synthesized using five plant extracts namely, *Syzygium cumini*, *Bauhinia variegata*, *Cedrus deodara*, *Lonicera japonica* and *Eleaocarpus sphaericus* as stabilizers and emulsifiers. The synthesis was done by sonicating poly(D,L-lactide) solution with dichloromethane and the aqueous plant extract. The transmission electron microscopy (TEM) analysis showed that the produced poly(D,L-lactide) NPs are spherical in shape and with different average sizes depending on each plant extract used. The poly(D,L-lactide) NPs from *Lonicera japonica* were uniform and therefore were selected for the loading of quercetin as a model for delivery of anticancer drug. The loading of quercetin was confirmed by the reduction of its absorption at 350 nm. The in vitro release study using phosphate buffer showed a burst release of around 20–27% of quercetin in 30 min, followed by controlled release of 32% after 24 h [48]. Similar study reported the green synthesis of PLGA NPs in a solvent-free method using castor oil derivative (acrysol oil). The study also incorporated resveratrol into the green PLGA-NPs which showed enhanced cytotoxicity against MCF-7 cells [49].

Mukherjee et al. (2012) used the aqueous extract of *Eclipta alba* to formulate spherical GNPs. The synthesis was optimized using different volumes of the plant extract. The GNPs showed efficient in vitro stability when incubated with different buffer and biological solutions. The biosafety of the GNPs (up to 114 µM) was shown by maintaining the viability of MCF-7 and MDA-MB-231 after 48 h incubation. However, 10 µM of the GNPs loaded with DOX exhibited 50% cell viability reduction of MCF-7 cells after the same period [50].

**Figure 4:**
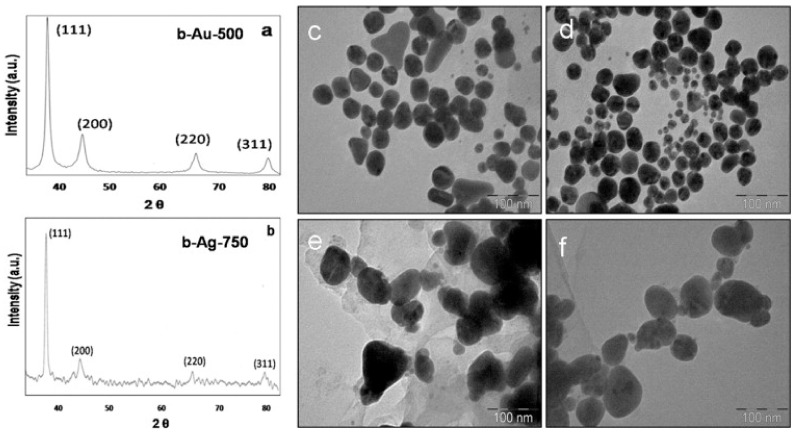
The characterization of the MNPs showing the X-ray diffraction peaks (**a**,**b**) confirming the crystallinity of the GNPs (denoted b-Au-500) and AgNPs (denoted b-Ag-750) from *B. monosperma*. c-f are the TEM images of the GNPs and AgNPs synthesized using different volumes of the *B. monosperma* extract 250 µL (**c**), 500 µL (**d**) for GNPs and 500 µL (**e**), 750 µL (**f**) for AgNPs. Reproduced from [51].

Biocompatible GNPs and silver nanoparticles (AgNPs) were synthesized using the leaf aqueous extract of *Butea monosperma*. The reaction was optimized by varying the leaf extract volume and keeping the volume of the gold and silver salts unchanged, which produced NPs after 5 min and 2 h for GNPs and AgNPs, respectively (Figure 4). The study reported the use of AgNO_3_ staining to confirm that low molecular weight proteins content of *B. monosperma* were responsible for the synthesis and stabilization of the produced MNPs. Dynamic light scattering (DLS) analysis confirmed the conjugation of DOX into the biosynthesized GNPs and AgNPs with the increase in their hydrodynamic size. Both MNPs loaded with DOX exhibited higher cytotoxicity compared to the pristine drug against mouse melanoma (B16F10) cells. Further, the fluorescence microscopy indicated higher red fluorescence in case of the DOX loaded into the MNPs compared to the free DOX, this proves higher internalization of DOX inside the B16F10 cells when loaded into the MNPs [51].

DOX was also loaded into GNPs biosynthesized using the aqueous extract of *Peltophorum pterocarpum*. The study explored the cytotoxicity of the loaded DOX in mice injected with B16F10 cells. The results displayed a substantial time-dependent reduction of the tumour volume in the mice treated with the loaded DOX-GNPs in comparison the free DOX treated mice [52].

The eggplant fruit extract was also reported in the reduction and stabilization of GNPs using the irradiation of natural sunlight. The produced GNPs were functionalized wit HA as a targeting agent. The carboxylic groups of HA were activated by 1-ethyl-3-(3-dimethylaminopropyl) carbodi-imide and N-hydroxysulfosuccinimide to allow its conjugation with the amid groups of the anticancer drug, metformin. The release of the loaded metformin was found to be negligible at higher pH, citing the targeting effect of the nanocarrier. Moreover, the loaded metformin showed higher cytotoxicity compared to the free drug. The MTT results after 48 h gave IC_50_ value of 4 µg/mL against HepG2 cells for the loaded metformin compared to 10 µg/mL for the free drug. The authors used the acridine orange and ethidium bromide staining experiment and showed that loaded metformin induce cell death via apoptosis. Interestingly, the same experiment showed no change on the viability of mouse embryonic fibroblast (NIH 3T3) cells, which was attributed to the targeting effect of HA to the cluster determinant receptor (CD-44) that is expressed on HepG2 but not on NIH 3T3 cell line [53].

Ganeshkumar et al. (2013) reported the use of fruit peel extract of *Punica granutum* (pomegranate) in the synthesis of stable GNPs. The authors coupled FA as targeting ligand and the coupling was confirmed by a red shift in the UV-Vis spectrum of the GNPs. After, 5-FU was loaded into the GNPs, which was shown by the quenching of the 5-FU fluorescence. It was also found that the nanocarrier was safe up to concentration of 750 µg/mL by evaluating the morphology, hatching and survival rate of the Zebrafish embryos. To utilize the targeting effect of FA against breast cancer, the cytotoxicity of nanocarrier loaded with the 5-FU was done against MCF-7 cell line and the MTT results gave IC_50_ value (250 ng/mL) three times lower than the IC_50_ value of the free 5-FU (1000 ng/mL). The Western blot analysis has confirmed that nanocarrier loaded with 5-FU induced apoptosis via G0/G1 cell cycle arrest [54]. The 5-FU loaded on GNPs formulated from the *Borassus flabellifer* fruit extract also showed higher cytotoxicity against the pancreas cancer MiaPaCa-2 cell line than free 5-FU as exhibited by the MTT assay [55].

Almond seed water extract was also used to biosynthesize GNPs. The biosynthesized GNPs were capped using polyethylene glycol 9000 (PG9) before the functionalization with quercetin as an anticancer drug model. The MTT results against MCF-7 showed that only the biosynthesized GNPs functionalized with PG9 and quercetin induced cell death, whereas quercetin alone and the green GNPs without PG9 or quercetin did not show significant antiproliferative activity [56].

The biosynthesis of AgNPs was also reported using the fruit water extract of the Indonesian *Garcinia mangostana*. The optimization was done by changing the concentration of AgNO_3_, temperature and the pH conditions. The loading of protocatechuic acid on the AgNPs resulted in significant antiproliferation activity (80%) against HCT116 colorectal cells compared to (5%) from the free AgNPs at concentration of 15.6 µg/mL. The IC_50_ of the free protocatechuic acid was 15 times higher than the IC_50_ of the loaded protocatechuic acid against the same cell line. The authors found that the cytotoxicity of the AgNPs loaded with protocatechuic acid was attributed to the loss of mitochondria’s membrane potential and the generation of ROS [57].

Cai et al. (2020) reported the synthesis of magnetic nanocomposites of Fe_3_O_4_ NPs facilitated by *Euphorbia cochinchensis* leaf extract. The resulted NPs were then incorporated with mesoporous silica and modified with carboxyl groups to prevent their agglomeration. The green nanocomposites were then loaded with DOX and showed high release at acidic pH. With the aid of external magnetic field, the authors concluded that these nanocomposites could be utilized for targeted and controlled release of DOX at tumour site [58].

The synthesis of ZnONPs was also facilitated by the tea ethanolic extract. The NPs were then loaded with chitosan to facilitate the targeting effect and were then loaded with paclitaxel. The nanocarrier showed selective toxicity against MCF-7 cells with minimum effect on normal fibroblasts compared to the free paclitaxel [59].

### 2.3. Plant Polyphenols and Pospholipids-Based Nanocarriers

Plant-based polyphenols are water soluble amphiphilic polymers which are found abundantly in nature. These polymers contain many phenolic hydroxyls in their structures that increase their reactivity towards several metal ions including Au^3+^, Ag^+1^, Fe^3+^, etc. [60,61,62]. Plant polyphenols are highly considered for the synthesis of MNPs for the following reasons: First, the reductive phenolic hydroxyls of plant polyphenols enhance the water-solubility of the MNPs and prevent their decomposition by oxygen atmosphere [63]. Secondly, the plant polyphenols protect the MNPs from aggregation due to the presence of hydrophobic and rigid backbones in their structures [64].

Jiang et al. (2019) exploited the reducing capabilities of green tea’s polyphenols to produce gold nanoclusters (GNCs) suitable for photothermal treatment. The clusters were loaded with DOX and showed enhanced cytotoxicity activity against B16F10 cells, in which the cell viability results recorded from MTT assay were 12.1 ± 3.1 and 14.3 ± 0.5% for the loaded and free DOX, respectively. However, the cell viability induced by the GNCs-DOX was reduced to 7.7 ± 0.1% when using laser at 808 nm [65].

Another study reported the biosynthesis of graphene oxide using tea polyphenols for the delivery of DOX. The biosynthesized graphene oxide led to enhanced cytotoxicity of DOX against human adenoid cystic carcinoma (ACC2) cell line by delivering it to the nuclei [66].

**Figure 5:**
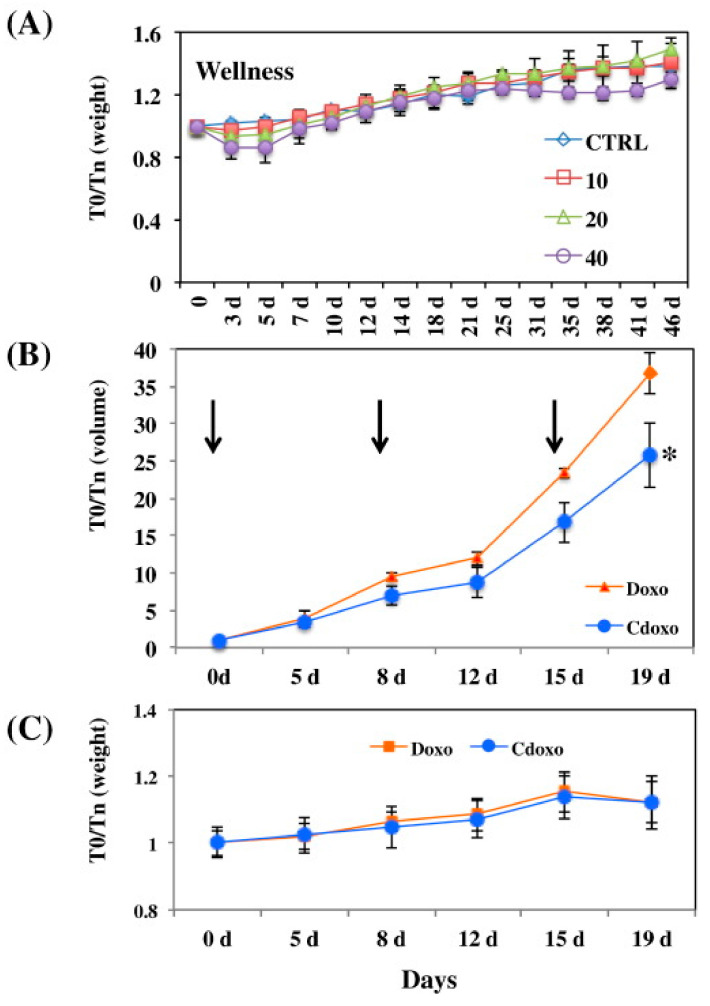
(**A**) The effect of different concentrations of CNPs loaded with DOX (in mg/mL) on mice weight at different time intervals. (**B**) The tumour volume reduction after treating the mice with 3 mg/mL of CNPs loaded with DOX (denoted Cdoxo) and free DOX (denoted Doxo), treatment times are indicated by the arrows. * *p* value < 0.05. (**C**) Weight of the treated mice with similar regimen as in (**B**). T0/Tn is the parameter at the zero time over the time on day as illustrated. Reproduced from [67].

Black tea polyphenols were also suggested to take a role in the synthesis of carbon nanoparticles (CNPs) as shown by the UV–Vis absorption and fluorescence spectra of the produced CNPs. The CNPs were found to escape the lysosomal degradation, which can assist in the delivery of drugs. The CNPs were then functionalized with DOX, which showed a reduction of tumour volume in vivo more than free DOX, with no negative effect on the mice health as indicated by their stable weight throughout the experiment (Figure 5) [67].

Resveratrol (3,4′,5-trihydroxy-*trans*-stilbene) is a natural polyphenol, which is extensively reported with anticancer activity [68,69,70]. The synthesis of GNPs from resveratrol as a nanocarrier was reported in a simple one pot method. The produced GNPs was mixed with aqueous solution of DOX for loading purposes. Testing the anticancer properties of the DOX-GNPs complex against two human cervical cancer cells (HeLa and CaSKi) showed reversal of chemoresistance towards the DOX treatment [71].

Furthermore, the amphiphilic nature of the plant-polyphenols make them suitable for protein binding [72]. For example, a targeting nanogel comprised of HA (to target CD44 receptor in cancer cells) and EGCG (to bind to protein) was assembled for the intracellular delivery of granzyme B. The study reported the increased cytotoxicity against CD44 presenting HCT-116 cells indicating the potential use of HA and EGCG as protein carriers for cancer treatment [73]. In a similar study, the self-assembly of EGCG and HA was also explored for the delivery of cisplatin. EGCG was used to encapsulate cisplatin via hydrophobic interaction, to augment its cytotoxicity and to reduce its side effects [74]. The affinity of EGCG to proteins enabled its conjugation with bovine serum albumin (BSA) to form a nanosphere intended for drug delivery of DOX [75]. The structure of BSA can interact with trypsin and glutathione, which are overexpressed by cancer cells [76,77], making the nanocarrier responsive to these biomarkers and increase its targeting effect.

Phospholipids are widely used raw materials for pharmaceutical products. They are included in several types of formulations, such as emulsions, micelles, suspensions, and liposomal preparations. These natural amphiphilic compounds are basic composition of biological membranes and are effective alternative to synthetic emulsifiers [78]. Phospholipids can be glycerophospholipids such as phosphatidylcholine, phosphatidylethanolamine, phosphatidylinositol and phosphatidylserine or can occur as sphingolipids [79]. Natural phospholipids can be isolated from dairy products and egg yolk, or from plant sources such as soybean and rapeseed lecithin and canola oil [80].

Liao et al. (2016) reported the use of EGCG in the preparation of nanoethosomes for transdermal delivery of docetaxel. The basic nanoethosomes were prepared using 2% soybean phosphatidylcholine, 30% ethanol, 1% Tween-80 and 0.1% sugar esters. The loading of 0.2% EGCG (*w*/*w*) into these nanoethosomes increased their stability. The EGCG-nanoethosomes also showed efficient skin penetration reaching the hypodermis, whereas the basic-nanoethosomes were only located in the dermis and epidermis as exhibited by confocal laser microscopy. Docetaxel was then loaded into the EGCG-nanoethosomes and its antitumor activity was evaluated against human melanoma cancer cells (A-375) implanted in mice skin. The EGCG-nanoethosomes loaded with docetaxel led to a significant reduction in the tumour size in comparison to the basic-nanoethosomes loaded with docetaxel [81].

The effectiveness of DOX was also enhanced by loading it on liposomes made of two layers of phospholipids of lecithin and *N*-trimethyl chitosan. The liposomes exhibited variable vascular targeting ability on human umbilical vein endothelial cells by altering the degree of quaternization of chitosan used. The loaded DOX also showed enhanced anticancer activity against hepatoma cells (H_22_) implanted on mice [82].

Zhang and co-workers (2017) developed liposomes made of phospholipids coating mesoporous carbon matrix for the oral delivery of the poorly soluble, docetaxel. The authors used carboxymethyl chitosan to protect the liposomes from the gastric pH. The incorporation of phospholipids and was found to increase the sustained release effects of the liposomes. Additionally, the carboxymethyl chitosan and phospholipids layers were shown to reduce the toxicity of mesoporous carbon matrix, which can lead to increased biocompatibility [83].

### 2.4. Microorganisms-Based Nanocarriers

The use of cells as drug delivery systems has been studied for their bioavailability, increased targeting effects, enhanced drug pharmacokinetics, controlled released capabilities and reduction of drug immunogenicity and toxicity [84]. Microbial based drug carriers are able to thrive under the hypoxic and acidic pH of the cancer tissue, making them ideal for targeted cancer therapy [85].

A study used yeast cells for the first time to host hydroxyapatite nanoscaffolds after the incubation with CaCl_2_ and Na_3_PO_4_ aqueous solutions. The yeast-hydroxyapatite nanoscaffolds were loaded DOX as a drug model and further functionalized with FA for targeting purposes (DOX-nHAP@yeasts-FA). The mice infected with hepG2 subcutaneously showed significant reduction in their tumour volume by DOX-nHAP@yeasts-FA as well as free DOX. However, the authors highlighted the safety of using DOX-nHAP@yeasts-FA because of their lower toxicity on normal cells in comparison to the free DOX [86].

**Figure 6:**
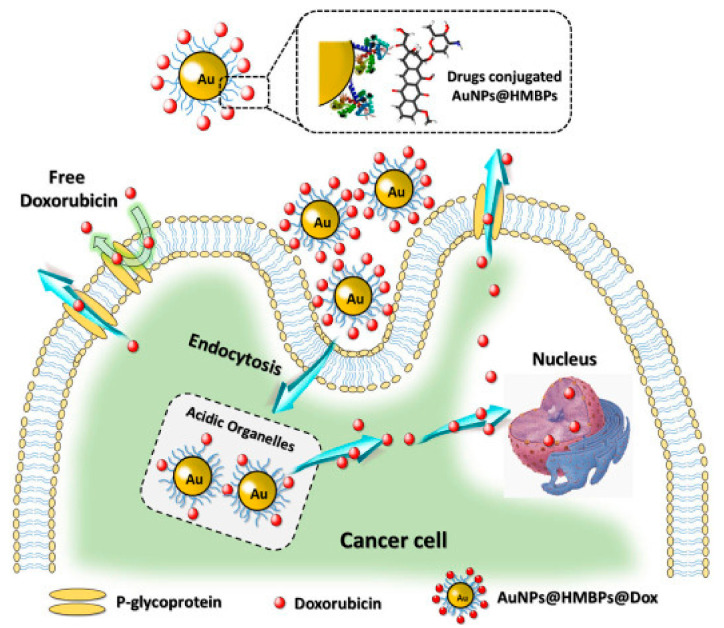
Synthesis of GNPs from HMBPs expressed by *E. coli* and subsequent loading of DOX and its release inside the cells in comparison the free DOX. Reproduced from [87].

The synthesis of GNPs was also reported from recombinant *E. coli* expressing heavy metal binding proteins (HMBPs). The biogenic GNPs were found to be safer than conventional synthesized GNPs. Further, the loading of DOX on the biogenic GNPs was done and demonstrated higher apoptosis rate than the free DOX mainly due to the improved intracellular uptake as reported by the authors (Figure 6) [87].

Similar to the plant extracts, the use of microbial culture to reduce metal salts and stabilize the produced MNPs has been reported. Kumar et al. (2014) reported the formation of spherical GNPs from the *Delftia* sp., which were loaded with resveratrol. The authors compared the cytotoxicity of the free and loaded drug against A549 cell line and reported 65% higher cytotoxicity in case of the loaded resveratrol. Furthermore, the release of resveratrol from the GNPs was significantly higher at acidic pH conditions, which shows the increased targeting effect from using these nanocarriers [88].

Table 1 lists the green components mentioned above. A summary of the different NPs formulations reviewed herein is included in Table 2.

## 3. Conclusions

Different green components were shown to be key players in the synthesis and in the function of different types of nanocarriers. The roles of these green components range from being a scaffold for materials such as drugs or targeting ligand, to the reduction of metal salts and the stabilization of MNPs. These green components were derived mainly from plant or microbial sources, which led to the biosynthesis of biocompatibility nanocarriers with improved safety margin that can allow their use in biological applications. Furthermore, the shape, size, and stability of green nanocarriers could be optimized by regulating the pH, reaction temperature, reactants’ concentrations, and time of incubation during the synthesis. In most of the reports, the green nanocarriers were mainly found to increase the anticancer activity of the loaded drugs. This was done by having augmented anticancer properties, by increasing the targeting effect, or by excreting controlled release. Some reports also formulated pH responsive nanocarriers to take advantage of the different pH levels between cancer and normal tissues, which can assist in increasing the target effects of their therapeutic payloads.

There are few challenges that face the potential use of green based nanocarriers in the market. Similar to other conventional nanomaterials, their synthesis remains complicated especially with polymeric NPs or liposomes. In addition, there are unsubstantial concerns of possible allergens or toxicity caused by impurities that can be found on the natural isolated components such as total plant extracts or natural phospholipids for example. However, using these natural parts can improve the safety of the nanomaterials. This is because biodegradable and biocompatible green components can reduce the toxicity that can arise from the nonbiodegradable residual parts of the conventional drug delivery systems. The green components can facilitate the synthesis without the need of toxic chemicals during the preparation of NPs, which improve the safety of the final product. Importantly, unlike the synthetic materials, these green renewable components will certainly reduce the cost of the upscaled production for commercial use.

## Figures and Tables

**Table 1:** Different types of green components and their functions as nanocarriers.

Examples of Green Component in Nanocarriers	Function
**Alginate**	Reduction of metal salts and stabilization of the formed MNPs
**CMC**	Improve drug entrapment Forms pH responsive nanocomposites
**CD**	Increased drug encapsulation High thermal and chemical stability
**Chitosan**	Can be protonated under acidic media to form pH-responsive hydrogels Can be attracted to negative charged tumour cells in drug delivery systems
**CR**	Interacts with cationic materials due to the presence of anionic sulphate groups Crosslinked with chitosan to prevent burst release of drugs from hydrogels
**Gum**	Reduction of metal salts and stabilization of the formed MNPs A scaffold for ligands and drugs
**HA**	Targeting agent against cancer cells with HA surface receptors
**MMt**	Adds mechanical strength to nanocomposites hydrogels Improve drug entrapment Enhance swelling property of hydrogels Control drug release
**Phospholipids**	Natural surfactant for preparation of liposomes Increase sustained release
**Plant extracts**	Reduction of metal salts and stabilization of the formed MNPs
**Pullulan**	Reduction of metal salts and stabilization of the formed MNPs

**Table 2:** Summary of the different types of reported green-based nanocarriers.

Formulation	Size (nm) *	Shape of the NPs/Formulation	Composition **	Drug Loaded	Advantage of Using the Nanocarrier	References
**Magnetic nanocomposite**	141 ± 6	Spherical and ellipsoidal shape	CR oligosaccharides/GNPs	Epirubicin	Increased cytotoxicity	[16]
	4 for the Fe_3_O_4_ NPs	Spherical Fe_3_O_4_ NPs	Fe_3_O_4_ NPs/CR/chitosan	Methotrexate	Controlled release	[18]
	9.2 ± 1.3 for Fe_3_O_4_ NPs (TEM)	Sheet-like matrix with spherical Fe_3_O_4_ NPs	CR/MMt/F_3_O_4_ NPs	Protocatechuic acid	Targeted release	[20]
	Not reported	Irregular and coarse nanogel with spherical Fe_3_O_4_ NPs	CR/m-MMt/chitosan/Fe_3_O_4_ NPs	Sunitinib	Controlled release	[19]
	<50 (TEM)	Dispersed	Fe_3_O_4_ NPs/*E. cochinchensis* leaf extract/mesoporous silica surface modification with carboxyl groups	DOX	Controlled and targeted release	[58]
**Nanocomposite**	6.4 nm in height (Atomic Force Microscopy (AFM))	Nanosheet	CD/graphene oxide sheets/hyaluronated adamantane	Camptothecin	Increased cytotoxicity and targeted release	[32]
	171.25 nm in height (AFM)	Nanosheet	Tea polyphenols/graphene oxide sheets	DOX	Increased cytotoxicity and target release	[66]
	17	Spherical	Black tea aqueous extract/CNPs	DOX	Increased cytotoxicity and target release	[67]
	202	Spherical	EGCG/BSA/FA	DOX	Increased cytotoxicity and target release	[75]
	225	Spherical	Sericin/rice bran albumin/gellan gum	DOX	Sustained and controlled release	[24]
	Variable sizes (70 ± 30 to 143 ± 36) depending on the plant extract used	Spherical	*S. cumini,**B. variegata, C. deodara, L. japonica and E. sphaericus*/poly(D,L-lactide) NPs	Quercetin	Controlled release	[48]
	375	Spherical	Acrysol oil/PLGA NPs	resveratrol	Increased cytotoxicity and sustained release	[49]
	120 ± 28 (DLS)	Spherical	Maltodextrin/gum arabic	EGCG	Increased cytotoxicity	[27]
	187 (DLS)	Well dispersed	Alginate-curcumin/GNPs	Methotrexate and curcumin	Increased cytotoxicity	[36]
	80 nm in height (AFM)	Spongy structure withsquare and lamellar shapes on the graphene sheets	CMC-Zinc metal framework/graphene oxide sheets	DOX	Controlled and sustained release	[43]
	4–5 mm for wet beads (visual)	Spherical	CMC/chitosan/ZnONPs	5-FU	Controlled release	[44]
	7–9 mm for wet beads (SEM)	Rough surface	CMC/layered double hydroxide (zinc/aluminum)	5-FU	Controlled release	[45]
	Variable according to type of chitosan used	Spherical	Lecithin/ *N* -trimethyl chitosan	DOX	Increased cytotoxicity and targeting release	[82]
**MNPs**	20–25 (TEM)	Spherical	Gum karaya/GNPs	gemcitabine hydrochloride	Increased cytotoxicity	[22]
	13 ± 1 (TEM)	Spherical	Gellan gum/GNPs	DOX	Increased cytotoxicity	[25]
	13 ± 5 (TEM)	Spherical	Porphyran/GNPs	DOX	Increased cytotoxicity and targeted release	[30]
	2–4 (TEM)	Spherical	Gum kondagogu/PtNPs	5-FU	Increased cytotoxicity and sustained release	[23]
	Variable sizes depending on incubation time and shape	Spherical/hexagonal/triangular	*E. alba *aqueous extract/GNPs	DOX	Increased cytotoxicity	[50]
	70.90 ± 8.42 (TEM)	Quasi spherical	Aqueous peel extract of pomegranate/GNPs/FA	5-FU	Increased cytotoxicity	[54]
	Variable sizes depending on the type of MNPs and their shapes	Spherical/hexagonal/triangular	leaf aqueous extract of *B. monosperma*/GNPs and leaf aqueous extract of *B. monosperma*/AgNPs	DOX	Increased cytotoxicity	[51]
	54.2 ± 2.1 (DLS)	Spherical	*Aqueuous leaf extract of P. pterocarpum*/GNPs	DOX	Increased cytotoxicity	[52]
	5–15 (TEM)	Spherical	*Almond seed water extract*/GNPs/PG9	Quercetin	Increased cytotoxicity	[56]
	55 ± 3 (DLS)	Spherical	eggplant fruit extract/GNPs/HA	Metformin	Increased cytotoxicity	[53]
	32.7 ± 5.7 (TEM)	Spherical	*G. mangostana fruit peel extract*/AgNPs	Protocatechuic acid	Increased cytotoxicity	[57]
	20.9 ± 4.4 (TEM)	Spherical	Resveratrol/GNPs	DOX	Increased cytotoxicity	[71]
	11.35 X-ray diffraction	Spherical	Tea ethanolic extract/ZnONPs/Chitosan	Paclitaxel	Targeted release	[59]
	11.3 (TEM)	Spherical	*Delftia* sp. culture broth/GNPs	Resveratrol	Increased cytotoxicity and targeted release	[88]
	5–20 (TEM)	Spherical	*E.**coli* expressing HMBPs/GNPs	DOX	Targeted release	[87]
	11 ± 5 (TEM)	Spherical	Pullulan/GNPs	Cassiarin A chloride derivatives	Increased cytotoxicity	[39]
	13.7 ± 1.9 (TEM)	Spherical	*para*-aminobenzoic acid-quat188-pullulan/GNPs	DOX	Increased cytotoxicity	[40]
	12.6 ± 1.5 (TEM)	Spherical	FA-*para*-aminobenzoic acid-quat188-pullulan/GNPs	DOX	Increased cytotoxicity	[41]
**Nanoclusters**	295.2 ± 7.7 (TEM)	Cluster	Green tea/GNCs	DOX	Increased cytotoxicity and targeted and controlled release	[65]
**Nanoethosomes**	72.4 ± 4.5 (TEM)	Smooth edges and compact	0.2% EGCG, 2% soybean phosphatidylcholine, 30% ethanol, 1% Tween-80 and 0.1% sugar esters	Docetaxel	Increased cytotoxicity	[81]
**Quantum dots**	4.9 ± 0.7 (TEM)	Spherical	CMC/(Cu-In-S/ZnS) quantum dots	DOX	Increased cytotoxicity and targeted and controlled release	[46]

* The characterization method is mentioned between brackets. ** The green components(s) are underlined.

## Abbreviations

5-FU—5-fluorouracil; A-375—human melanoma cancer cells; A549—human lung cancer cells; ACC2—human adenoid cystic carcinoma cell line; AFM—atomic force microscopy; AgNPs—silver nanoparticles; BSA—bovine serum albumin; C6—rat glioma cell line; CMC—carboxymethylcellulose; CD—cyclodextrin; CNPs—carbon nanoparticles; CR—carrageenan; DLS—dynamic light scattering; DOX—doxorubicin; DU145—human androgen independent prostate cancer cells; EGCG—epigallocatechin-3-gallate; Fe_3_O_4_ NPs—iron oxide nanoparticles; FA—folate; GNCs—gold nanoclusters; GNPs—gold nanoparticles; HA—hyaluronic acid; HCT-116—human colon cancer cells; HepG_2_—human liver cancer cells; HMBPs—heavy metal binding proteins; K562—human blood cancer cell line; KATO-III—human gastric carcinoma; MCF-7—breast cancer cells; MDA-MB-231—human breast cancer cells; m-MMt—magnetic montmorillonite; MMt—montmorillonite; MNPs—metallic nanoparticles; NIH 3T3—mouse embryonic fibroblast; NPs—nanoparticles; PEG—polyethylene glycol; PG9—polyethylene glycol 9000; PtNPs—platinum nanoparticles; TEM—transmission electron microscopy; ZnONPs—zinc oxide nanoparticles.
